# Challenges and Progress in Neurosurgery: A Comprehensive Assessment of the Landscape in Nepal

**DOI:** 10.7759/cureus.73566

**Published:** 2024-11-12

**Authors:** Ganesh Phayal, Pragya Rijal, Sabrina Muhanna, Ernest Barthelemy, Amrit Chiluwal

**Affiliations:** 1 Neurosurgery, SpineCare Long Island, Long Island, USA; 2 Neurosurgery, State University of New York (SUNY) Downstate Health Sciences University, Brooklyn, USA; 3 Neurosurgery, Bir Hospital, Kathmandu, NPL; 4 Neurosurgery, Jamaica Hospital Medical Center, Queens, USA

**Keywords:** global burden of disease, global neurosurgery, lmic, nepal, neurosurgery in nepal, wfns, who

## Abstract

A large portion of the global population lacks access to essential surgical care, despite surgical conditions contributing significantly to the overall burden of disease worldwide. This burden is heaviest in developing countries like Nepal, where disparities in access to surgery for neurosurgical disorders stem from health system failures including an inadequate neurosurgical workforce, lack of requisite neurosurgical equipment, and challenges in perioperative care. By bibliometric analysis of neurosurgery-related publications in Nepal through PubMed search using the search terms “Neurosurg* AND Nepal”, we have found a total of 528 articles with a notable increase in the number of such publications from 14 to 25 between 2015 and 2016, with over 300 published in 2021, 2022, and 2023. Nepal has also seen a drastic increase in neurosurgeon density from 0.166 per 100,000 population in 2016 to 0.377 per 100,000 population in 2023 compared to 0.780 in China, 0.274 in India, 0.130 in Bhutan, and 0.092 per 100,000 population in Bangladesh in 2023. This study underscores the pressing need for enhanced neurosurgical capacity and improved health equity in Nepal.

## Introduction and background

Despite the growing global burden of surgical disease and concomitant need for essential surgery, most people worldwide still lack access to safe, timely, affordable, and high-quality surgical care. Global surgery has gained widespread recognition as a vital component of global public health, especially in light of the recent comprehensive report by the Lancet Commission on Global Surgery which highlights regions of the world that are most affected by an unmet need for essential surgical and anesthesia care, such as low-and middle-income countries (LMICs) of South Asia, like Nepal [1]. Access to surgical subspecialty care in Nepal, such as neurosurgery, is a challenge due to affordability, accessibility, and/or fear [2].

While prior literature has discussed the early era of neurosurgery in Nepal, this article offers a comprehensive review of its current status, focusing on the country’s unique social, political, and economic context within South Asia and providing updated information on the field. As a developing nation, Nepal serves as a representative example, highlighting the struggles for healthcare equity experienced across diverse settings. Following brief background data and biographical sketches of key figures in Nepalese surgery and neurosurgery, we present a needs assessment of neurosurgery in Nepal, compiling relevant, publicly available data on the country’s neurosurgical disease burden and healthcare delivery capacity. The article concludes with recommendations for researchers and community leaders to guide future contributions and foster collaborations.

**Figure 1:**
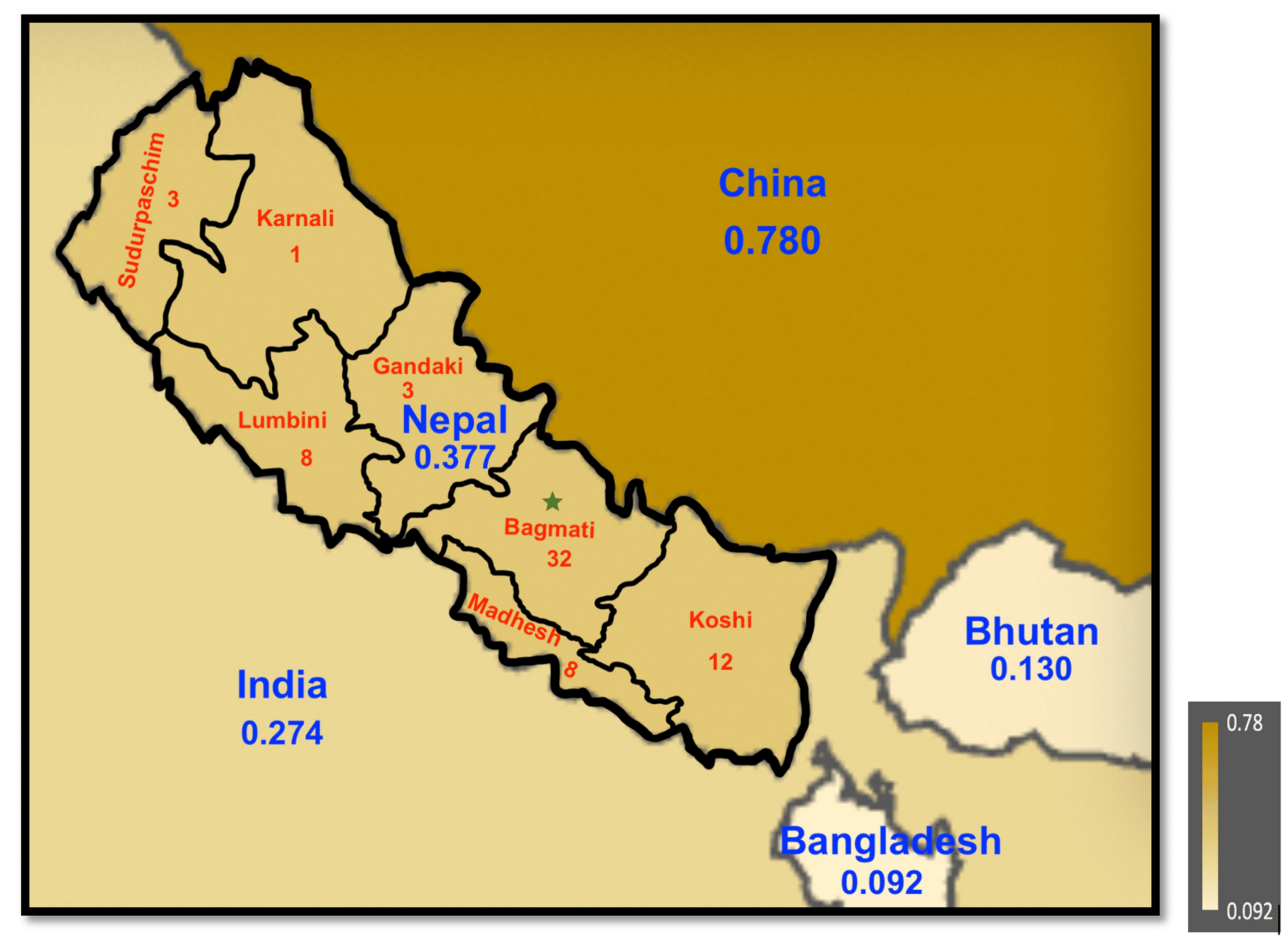
Density of neurosurgeons per 100,000 population of Nepal and its neighboring countries in 2023. The numbers in red represent the neurosurgical facilities distribution within the seven provinces in Nepal. The green asterisk represents the province where the capital city, Kathmandu is located. The image was created using a free online tool from the website www.mapchart.net

Nepal is a landlocked country in South Asia, situated between China and India, and home to eight of the world’s ten highest peaks, including Mount Everest, the world’s tallest mountain, which shares a border with China. The country covers an area of 56,827 square miles and had an estimated population of around 30.7 million in 2022 (Figure 1). Governed by a federal parliamentary republic system, Nepal had a fertility rate of approximately 1.9 in 2022, with the population growing at a rate of 0.8% that year [3]. The death rate was recorded at 6.7 per 1,000 people in 2022. According to the World Bank database, the availability of hospital beds was 0.3 per 1,000 people as of 2012, with a median life expectancy at birth of 69.7 years for men and 73.9 years for women as of 2017 [4,5].

The history of neurosurgery in Nepal dates back to 1962, when Dr. Dinesh Nath Gongal, a general surgeon, performed the first intracranial surgery for pituitary adenoma. However, it was not until 1993 that Dr. Upendra Devkota formally established neurosurgical services in Nepal, marking the beginning of the modern era of neurosurgery. Dr. Devkota received his training at the Glasgow Institute of Neuroscience in the United Kingdom. Despite facing numerous challenges in setting up neurosurgical services in Nepal, Dr. Devkota's efforts succeeded with the support of Dr. Merwyn Bagan, MD, at the Institute of Medicine (IOM), Tribhuvan University & Teaching Hospital (TUTH). He also trained Dr. Mohan Sharma, who now serves as the current chair of neurosurgery at the same institution. This foundation has paved the way for the continued development of neurosurgical care in Nepal [6].

Although available in the country, specialized neurosurgical services were centralized in the capital city of Kathmandu for many years, thereby limiting access for people outside the city. Clinics in Kathmandu could only address common travel-related medical complaints; more serious illnesses often required referral to medical facilities in nearby cities such as New Delhi, Singapore, or Bangkok [7,8].

Neurosurgical services in Nepal eventually began to decentralize across the nation, with services being incorporated into institutions such as the BP Koirala Institute of Health Sciences (BPKIHS) in Dharan, Neurocardio Multispecialty Hospital in Biratnagar, Manipal Medical College & Teaching Hospital in Pokhara, and Nobel Medical College Teaching Hospital in Biratnagar [7,9]. Despite this expansion, neurosurgical care in Nepal still faces challenges due to limited resources and facilities compared to Western standards. The country’s medical facilities often grapple with resource shortages, and emergency medical services do not meet the quality levels found in developed countries like the United States.

## Review

Methods

We aimed to comprehensively assess the landscape of neurosurgery in Nepal, including publication output, the neurosurgical workforce, the availability of medical equipment, and the burden of neurosurgical diseases, using a combination of ecological studies and database queries from publicly available sources. To characterize the quantity and publication trends of neurosurgery-related literature about Nepal, we conducted a literature review using the search term 'Neurosurg* AND Nepal' and performed a bibliometric analysis of the results. For comparison, we used similar search terms for neighboring countries, such as 'Neurosurg* AND China,' 'Neurosurg* AND India,' 'Neurosurg* AND Bhutan,' and 'Neurosurg* AND Bangladesh,' and presented the data through graphic representation.

To evaluate the neurosurgical workforce in Nepal, we obtained data from the World Federation of Neurosurgical Societies (WFNS) workforce map. We benchmarked the data from Nepal geographically and socioeconomically by comparing our findings to data from neighboring countries categorized as either lower-middle or upper-middle income according to the World Bank classification.

To investigate the availability of medical equipment and healthcare facilities in Nepal, we queried the 2022 Global Atlas of Medical Devices (GAMD). Subsequently, we reviewed prior peer-reviewed and grey literature to identify additional sources of information on medical equipment and healthcare facilities in Nepal. Finally, we analyzed publicly available data from the World Bank’s DataBank and the Global Burden of Disease (GBD) database of the Institute for Health Metrics and Evaluation (IHME) to assess Nepal’s burden of neurosurgical diseases.

Results

Neurosurgical Research in Nepal

**Figure 2:**
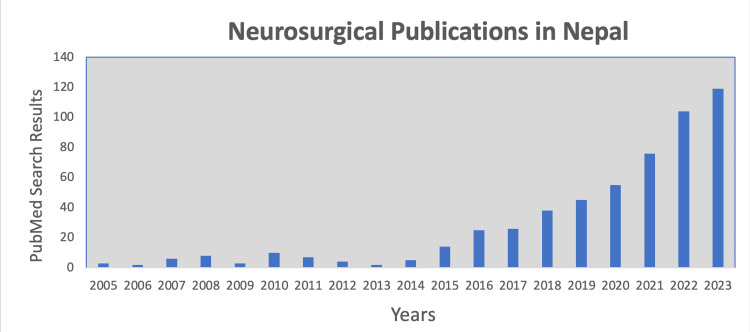
A bar graph showing the number of PubMed search results by year, at the time of writing, for the search term “Neurosurg* AND Nepal” from 2005 to 2023.

Our PubMed search for neurosurgery-related publications about Nepal yielded a total of 528 publications until December 2023. In comparison, similar searches for the four neighboring countries, China, India, Bangladesh, and Bhutan, yielded 53,146, 13,549, 330, and four neurosurgery-related publications, respectively. The number of articles addressing neurosurgery-related topics about Nepal has shown a notable increase over time. In 2015, there were 14 published articles on neurosurgery-related topics, which nearly doubled to 25 in 2016. In 2005, only three neurosurgery-related articles about Nepal were available in PubMed, but this count has steadily risen to 104 in 2022 and 119 in 2023 (Figure 2). This upturn in publications can be attributed, at least in part, to the influence of the report published by the Lancet Commission on Global Surgery [1]. The rise in scholarly output signifies a growing interest and attention toward academic neurosurgery in Nepal, demonstrating an increasing recognition of the field and its importance to the country.

Neuroimaging and Neurosurgical Facilities

As of 2022, the GAMD reports 11 radiotherapy apparatuses, 699 health posts, 201 health centers, and 65 general and/or district hospitals available in the country. In comparison, India has 979 pieces of radiotherapy equipment, China has 1,818 radiotherapy units, and Bhutan has one MRI machine, one CT scan machine, 492 health posts, 12 health centers, 10 general and/or district hospitals, and only one specialized hospital (Table 1) [10]. Despite the data from the GAMD, a recent literature review indicates that more advanced equipment, such as neuronavigation, intraoperative magnetic resonance imaging, O-arm, Cavitron, and ultrasonic surgical aspirator, as well as facilities required for radiosurgery and functional neurosurgery, are available in some centers in Nepal [6]. The discrepancies between the equipment reported by the GAMD and those mentioned in prior literature highlight issues with health system reporting and policy failures. Nonetheless, it is important to note that the availability of these services is limited to certain geographic locations, making access a privilege for many. Financial barriers remain a major obstacle for patients seeking neurosurgical care in Nepal, where the cost of frequent imaging is prohibitive for many individuals. However, the government of Nepal provides support of USD 1,000 for patients with head injuries, spinal injuries, and brain tumors [6].

**Table 1:** Distribution of radiotherapy equipment, specialized and/or regional hospitals in Nepal and its neighboring countries.

Country	World Bank Income Level	Population (in Millions, approximate)	Quantity of radiotherapy apparatuses	Specialized/regional/national hospitals
Bangladesh	Lower-Middle Income	165	43	N/A
Bhutan	Lower-Middle	0.78	0	1
China	Upper-Middle	1439	1818	N/A
India	Lower-Middle	1380	979	N/A
Nepal	Lower-Middle Income	30	11	12

Moreover, the Institute of Neuroscience in Biratnagar offers skull-based and cerebrovascular training and fellowships to many young neurosurgeons and residents. This newly established institute has state-of-the-art facilities, including an intraoperative 3T magnetic resonance imaging (MRI) machine, an O-arm machine, and a visible microscope that allows for performing complex microsurgical procedures. Additional training centers and surgical equipment contribute toward achieving the recommended neurosurgical density ratio as well as improving outcomes for patients [6,11,12].

Neurosurgical Disease Burden in Nepal

**Figure 3:**
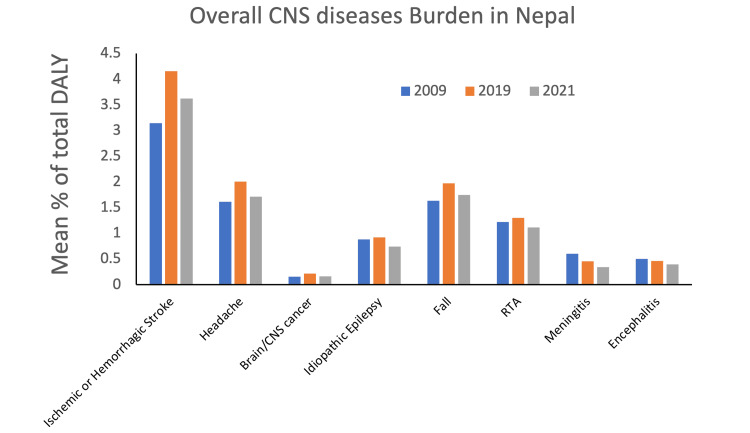
Mean percentage of total DALY representing overall CNS disease burden in Nepal in 2009, 2019, and 2021. DALY: Disability-Adjusted Life Years; CNS: Central Nervous System

There are a limited number of studies that have conducted epidemiological assessments of neurologic and neurosurgical disease burden in Nepal. The lack of data collection systems and resources in the country further compounds the challenge of assessing the incidence and prevalence of neurosurgical diseases. Since 2009, Nepal has not significantly reduced the burden of CNS diseases, with the highest prevalence seen in vascular CNS pathology (Figure 3). Existing literature indicates that stroke, meningitis, encephalitis, and metastatic brain cancers are the leading causes of disability-adjusted life years (DALY) [1,6].

**Figure 4:**
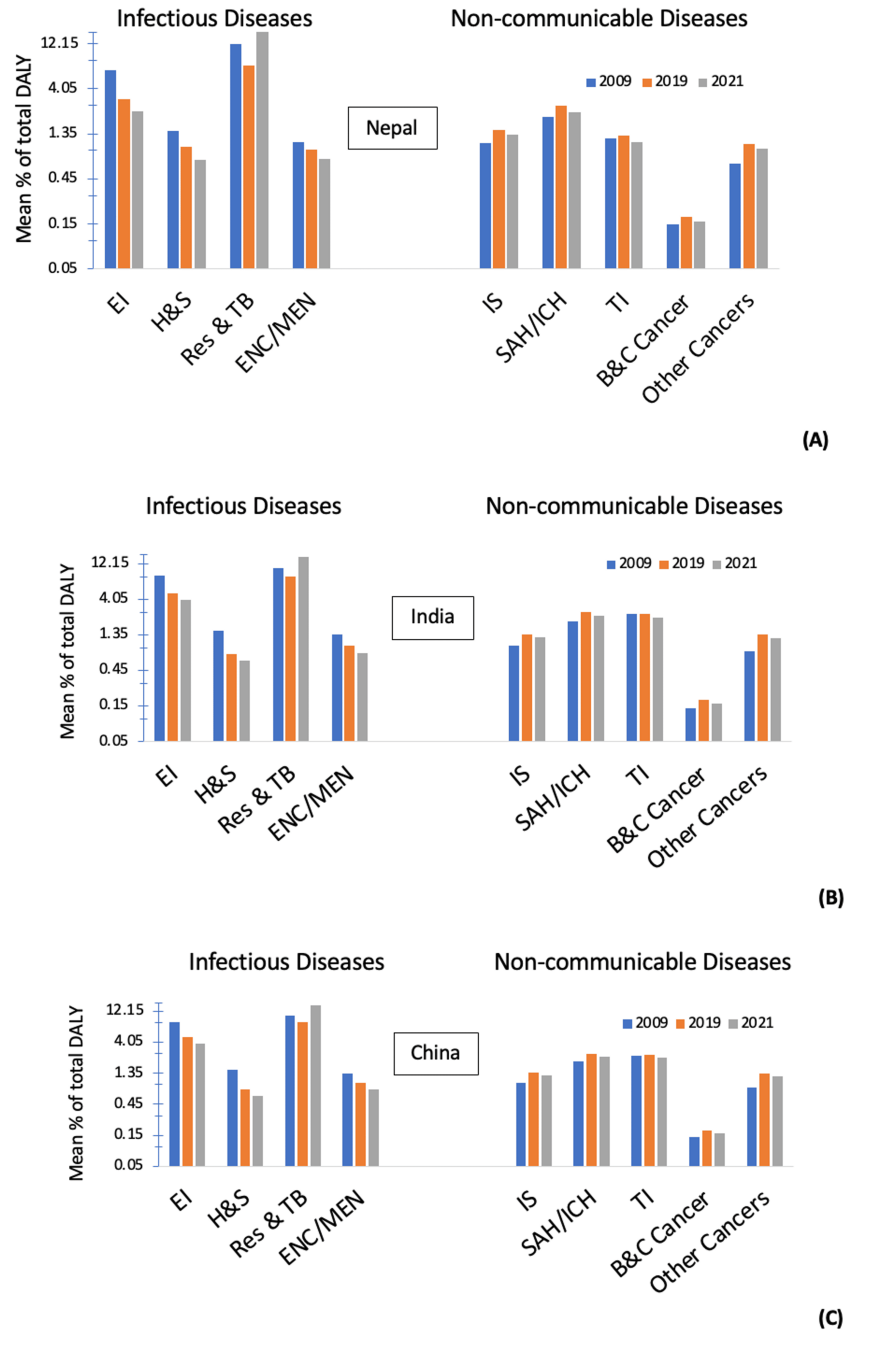
Mean percentage of total DALY among selected infectious and non-communicable diseases representing the burden of disease in 2009, 2019, and 2021 in (A) Nepal, (B) India, and (C) China. The other cancers include the combined data of malignant skin melanoma, tracheal, bronchus and lung cancer, breast cancer, kidney cancer, colon cancer, and rectal cancer. EI: Enteric Infections; H&S: HIV and Sexually Transmitted Infections; Res & TB: Respiratory infections and Tuberculosis; ENC/MEN: Encephalitis/ Meningitis; IS: Ischemic Stroke; SAH/ICH: Subarachnoid Hemorrhage/Intracerebral Hemorrhage; TI: Transportation Injury; B&C cancer: Brain and Central Nervous System Cancer

In 2021, deaths from non-communicable diseases in Nepal accounted for 53.87% of total deaths, while injuries and COVID-19 pandemic-related outcomes accounted for approximately 6% and 7.4% of total deaths, respectively. Non-communicable diseases accounted for 53.6% of total DALY, whereas communicable, maternal, neonatal, and nutritional diseases accounted for 34.14% of total DALY. Additionally, injuries contributed to 7.75% of total DALY, while COVID-19 pandemic-related outcomes accounted for about 4.5% of total DALY (Figure 4) [13].

Between 2009, 2019, and 2021, Nepal, India, and China have undergone similar epidemiological transitions, as indicated by the mean percentage of total DALY (Figure 4). The burden of neurosurgical diseases in Nepal is higher in areas outside Kathmandu, primarily due to a lack of appropriate medical equipment, healthcare professionals, and healthcare facilities. Emergency neurosurgical cases have a high mortality rate, and many patients do not survive the trip to the hospital, whether in Kathmandu or various cities in India. For the few survivors, there is a significant financial, social, and psychological burden on their families due to the changes in language, culture, and professional attitudes when seeking treatment in another country like India [1]. In the past, medical treatment was confined to small clinics and government-operated health centers until the establishment of neurosurgical services at the BP Koirala Institute of Health Sciences (BPKIHS) in 2006 [6,11].

Nepali Neurosurgical Workforce

Over the past decade, the number of neurosurgeons in Nepal has increased rapidly. In 2006 and 2008, Dr. Andrea from Russia and Dr. Iype Cherian, respectively, began providing neurosurgery services outside the capital city of Kathmandu. As of 2023, there were 116 neurosurgeons for a population of 30.7 million, resulting in a neurosurgeon density of 0.377 per 100,000 people. This represents a significant improvement from the 0.166 per 100,000 reported in 2016 by the WFNS workforce map (Figure 1). While Nepal's neurosurgeon density has approximately doubled from 2016 to 2023, the densities in neighboring countries have either remained the same or decreased [6,14] (Figure 1). The 2023 neurosurgeon densities in Bangladesh, Bhutan India, and China were 0.092-, 0.130-, 0.274-, and 0.780 per 100,000 population respectively [15-17].

Despite progress in increasing the number of neurosurgeons in Nepal, a shortage of 250 neurosurgeons persisted until 2019, preventing the country from reaching the recommended ratio of one neurosurgeon per 100,000 people. Although approximately 20 to 25 neurosurgery graduates emerge each year, there remains an uneven distribution of neurosurgeons across the country. Over 40% of neurosurgeons are concentrated in Kathmandu, which serves only 14% of the nation’s population. This disparity highlights the need for a more equitable distribution of neurosurgical services throughout Nepal [6,18]. Additionally, until 2019, there were no formal neuroanesthesia or neurocritical care training programs in Nepal. In most hospitals, neurosurgical anesthesia and neurocritical care are provided by anesthesiologists with limited or no formal subspecialty training.

Discussion

Unmet Demand for Neurosurgical Care

The limited availability of reliable data on Nepal’s neurosurgical epidemiology highlights a pressing unmet demand for neurosurgical care. Despite recent growth in the field, substantial efforts are still needed to ensure the provision of fundamental and safe surgical care. Although Nepal has seen a significant increase in the number of newly trained neurosurgeons, the uneven distribution of these specialists between major urban centers and rural areas presents a barrier to accessing neurosurgical services [6,12].

On a global scale, there are about 73,000 neurosurgeons, resulting in an index of 0.93 per 100,000 population. In comparison, the USA has approximately 5,400 neurosurgeons, with 4,000 being board-certified, yielding an index of 1.6 per 100,000 population. Despite these figures, there is a pressing need for an additional 23,000 neurosurgeons to address at least five million essential, treatable neurosurgical cases annually in LMICs that go unmet each year [1,12,19,20].

Given that countries like Nepal bear a disproportionate burden of neurosurgical diseases, a rapid expansion of newly trained neurosurgeons is necessary to meet the estimated volume of cases. Presently, the country produces approximately 20-25 new neurosurgeons annually, but the unequal allocation across different regions highlights an ongoing unmet need for surgical care [6].

Budget Allocation and Healthcare Resources

The government plays a crucial role in the distribution and management of healthcare resources and budgets in Nepal. In 2020, healthcare expenditure accounted for 5.42% of Nepal's gross domestic product (GDP), reflecting an increase from 4.05% in 2004 [5,20]. Over less than two decades, total health spending surged from NPR 16.2 billion in 2000 to NPR 161 billion in 2017, representing an 894% increase and a compound annual growth rate of 14.4%. During this period, inflation averaged around 8%, indicating a real growth in health expenditures of approximately 6% per year [4,5,21]. The Nepal Health Sector Support Program provides the framework to ensure Nepal’s commitment to achieving Universal Health Coverage and the Sustainable Development Goals 3 by 2030 [21]. As of 2023, Nepal had a total of 14,313 healthcare facilities, including 215 public hospitals, but only 67 facilities offered neurosurgical care. Furthermore, the distribution of neurosurgical facilities across provinces is uneven, with Bagmati Province alone accounting for approximately 50% of the country's neurosurgical facilities (Figure 1) [4,6,22].

Financial Hardships and Health Financing

It is estimated that 48%-69% of households in Nepal face financial hardships and potential impoverishment due to healthcare expenses. To achieve long-term financial protection against the risk of catastrophic health expenditure at the household level, Nepal could implement a tax-based health financing system, a social health insurance scheme, or a combination of both. The country has committed to achieving universal health coverage (UHC) by 2030. International aid constitutes nearly 50% of Nepal's health budget, yet traditional healers such as Dhamis and Guruwas are still sought by over 50% of the Nepali population for initial healthcare needs [21]. The use of Dhamis' services is positively associated with increasing age, lower education levels, and adherence to Buddhism and Hinduism. These services show no significant association with gender, family income, or the type of disease (communicable or non-communicable), highlighting the urgency of improving public awareness, health education, and overall literacy [23].

Strategic Healthcare Spending and Infrastructure

Healthcare funding in Nepal has not reached optimal levels, and the COVID-19 pandemic has further highlighted the issue of neglected healthcare. Consequently, the government has taken steps to increase medical care funding, which has partially contributed to strengthening the existing neurosurgical infrastructure [6]. However, strategic healthcare spending is imperative to address the current surge in surgical disease burden. While infectious diseases pose a significant risk and contribute to the nation’s disease burden, the surge in surgical disease burden and the persistent neglect of surgical care funding demand urgent attention. Adequate training and distribution of neurosurgeons, improved healthcare financing systems, and strategic allocation of resources are essential to meet the growing demand for services and achieve universal health coverage in the country.

## Conclusions

The landscape of neurosurgery in Nepal reflects both challenges and progress in addressing healthcare disparities. This review provides updated information about the current state of neurosurgery in Nepal and compares it to that in neighboring countries. Although the field of neurosurgery is expanding in Nepal, particularly with the rapid growth in academic neurosurgery, the burden of neurosurgical diseases underscores the urgency of addressing gaps in data collection and resources. The rapid increase in the neurosurgical workforce is promising, but efforts are needed to ensure equitable distribution and meet the unfulfilled need for surgical care nationwide.
